# Validation of a Latin-American Spanish version of the Body Esteem Scale for Adolescents and Adults (BESAA-LA) in Colombian and Nicaraguan adults

**DOI:** 10.1186/s40337-023-00942-5

**Published:** 2023-12-08

**Authors:** Fabienne E. Andres, Tracey Thornborrow, Wienis N. Bowie, Ana Maria Chamorro, Gisell De la Rosa, Elizabeth H. Evans, Laura S. Fontalvo Acuña, David R. Kolar, Moises R. Mebarak, Juan Camilo Tovar Castro, Lynda G. Boothroyd

**Affiliations:** 1 Department of Psychology, Upper Mountjoy, South Road, Durham, DH1 3LE UK; 2 School of Psychology, University of Lincoln, Lincoln, UK; 3 Universidad de Las Regiones Autónomas de La Costa Caribe Nicaragüense (URACCAN), Bluefields, Nicaragua; 4 Department of Psychology, Universidad del Norte, Barranquilla, Colombia; 5 Department of Psychology, University of Regensburg, Regensburg, Germany; 6 Medical School, Pontificia Universidad Javeriana, Bogotá, Colombia; 7 Department of Psychology, Corporación Universitaria Reformada, Barranquilla, Colombia

**Keywords:** Body image, Body Esteem Scale for Sdolescents and Adults, Body satisfaction, Validation, Factor structure, Spanish, Latin America, Colombia, Nicaragua

## Abstract

**Background:**

Body dissatisfaction (BD) is a growing concern in Latin America; reliable and culturally appropriate scales are necessary to support body image research in Spanish speaking Latin American countries. We sought to validate a Latin-American Spanish version of the Body Esteem Scale for Adolescents and Adults (BESAA; Mendelson et al. 2001).

**Methods:**

The BESAA was translated, culturally adapted, and validated in a sample of adults in Colombia (*N* = 525, 65% women, *M*_age_ 24.4, *SD* = 9.28). We assessed factor structure (using confirmatory factor analysis (CFA), exploratory factor analysis (EFA) and exploratory structural equation model (ESEM)), internal reliability (using Cronbach’s alpha and omega), validity (using the Body Appreciation Scale BAS and Sociocultural Attitudes Towards Appearance Questionnaire SATAQ), test–retest stability in a small subsample (*N* = 84, using Intraclass correlations ICC) and measurement invariance across gender. To evaluate the generalizability of the scale, we assessed reliability, validity, and factor structure in a second sample from rural Nicaragua (*N* = 102, 73% women, *M*_age_ 22.2, *SD* = 4.72), and assessed measurement invariance across Nicaraguan and Colombian participants.

**Results:**

The scale showed good internal reliability and validity in both samples, and there was evidence of adequate test–retest stability in the Colombian sample. EFA showed a three-factor structure with subscales we labelled ‘*appearance-positive’*, ‘*appearance-negative’* and ‘*weight’*, that was confirmed using CFA and ESEM in the Colombian sample. Measurement invariance was confirmed across the Colombian and Nicaraguan samples, and across gender within the Colombian sample.

**Conclusion:**

The Latin-American Spanish version of the BESAA (BESAA-LA) appears to be a psychometrically sound measure with good reliability, validity and invariance across gender and countries. These results support the use of this scale to measure body satisfaction/dissatisfaction in Latin American adult populations.

**Supplementary Information:**

The online version contains supplementary material available at 10.1186/s40337-023-00942-5.

## Abstract (plain language summary)

Body dissatisfaction is a risk factor for eating disorders, so it is important to have measures to reliably assess body satisfaction/dissatisfaction. As most research is conducted in English-speaking countries, relatively few questionnaires have been translated to other languages. Here, we assessed the usefulness of a Latin-American Spanish translation of the Body Esteem Scale for Adolescents and Adults (BESAA), used to assess body satisfaction/dissatisfaction, in adult samples from two Latin American countries, Colombia and Nicaragua. Our results indicate that the Latin-American Spanish version of the BESAA (BESAA-LA) is a reliable and valid measure of body satisfaction/dissatisfaction in Colombian and Nicaraguan adults.

**Supplementary Information:**

The online version contains supplementary material available at 10.1186/s40337-023-00942-5.

## Resumen

**Introducción:**

La insatisfacción corporal (BD) es una preocupación creciente en Latinoamérica; cuestionarios confiables y culturalmente apropiados son importantes para fomentar la investigación de la imagen corporal en los países latinoamericanos. Tratamos de validar una versión en español latinoamericano de la Escala de Estima Corporal para Adolescentes y Adultos (Mendelson et al., 2001).

**Métodos:**

La escala fue traducida, adaptada culturalmente y validada en una muestra de adultos en Colombia (*N* = 525, 65% mujeres, *M*_*edad*_ 24,4, *SD* = 9,28). Se evaluó la estructura factorial (mediante análisis factorial confirmatorio (AFC), análisis factorial exploratorio (AFE) y modelo exploratorio de ecuaciones estructurales (ESEM)), confiabilidad interna (utilizando alfa de Cronbach y omega), validez (utilizando la Body Appreciation Scale BAS y Sociocultural Attitudes Towards Appearance Questionnaire SATAQ), estabilidad test-retest en una pequeña submuestra (*N* = 84, usando correlaciones intraclase ICC) e invarianza de medición entre géneros. Para evaluar la generalización de la escala, se evaluó la confiabilidad, validez y estructura factorial en una segunda muestra de adultos Nicaragüenses (*N* = 102, 73% mujeres, *M*_edad_ 22,2, *SD* = 4,72).

**Resultados:**

La escala evidenció una buena confiabilidad interna y validez en ambas muestras y mostró evidencia de una estabilidad temporal adecuada en la muestra colombiana. La EFA evidenció una estructura de tres factores con las subescalas que denominamos ‘*apariencia-positiva*’, ‘*apariencia-negativa*’ y ‘*peso*’ que se confirmó mediante AFC y ESEM en la muestra colombiana. Se confirmó la invarianza de medición en las muestras colombiana y nicaragüense, así como entre los géneros dentro de la muestra colombiana.

**Conclusión:**

La versión en español latinoamericano BESAA-LA es una medida psicométricamente sólida con buena confiabilidad, validez e invarianza entre géneros y países. Estos resultados apoyan el uso de esta escala para medir la satisfacción/insatisfacción corporal en poblaciones adultas latinoamericanas.

## Introduction

Body dissatisfaction (BD), defined as the discrepancy between the ideal and actual body [11], has been reported to occur in many Latin American populations [9, 22, 47, 71, 75]. BD is associated with an increased risk of obesity [59], low self-esteem [16], depression, and eating disorders [53, 66, 76]. BD also increases the likelihood of considering plastic surgery [50, 63, 68]. Recent reports placed Brazil, Mexico, and Argentina among the top ten economies with the highest prevalence of plastic surgeries performed worldwide. Colombia was ranked as 13th (International Association for Aesthetic/Cosmetic surgery survey 2020). Recent meta-analyses show high prevalence of eating disorders in Latin America, especially binge-eating disorder [36, 37]. However, Latin American research is dominated by studies from Mexico and Brazil and less is known about body image in other Latin American countries [39]. Furthermore, body image studies in Latin American contexts tend to rely heavily on a few scales that measure a limited range of constructs and that have been developed and/or translated for European Spanish populations. For instance, amongst the most widely used scales are the Spanish Body Shape Questionnaire [18, 58] and Figure Rating scales (e.g., Stunkard's Silhouettes Scale, [67]. Both mostly focus on the perceptual aspect of BD, neglecting other aspects of the multidimensional construct of body image (e.g., cognitive, emotional, and behavioural aspects; [10].

Efforts are underway to address the lack of suitable instruments for Latin American contexts. For instance, Gongora et al. 26, demonstrated sound psychometric properties and invariance in male and female adolescents in three countries in their validation of a Latin Spanish version of the Body Appreciation scale (BAS-2; [72]). Similarly, Mebarak Chams [46] translated and validated the Body Image State Scale (BISS; [12]) in a Colombian sample and found similar factor structure and reliability to the original. Lacking, however, is a linguistically and culturally appropriate scale that includes positive and negative aspects of body image, validated for use in Latin American populations.

### The Body Esteem Scale for Adolescents and Adults (BESAA)

The current study sought to address this gap by translating, culturally adapting, and validating the Body Esteem Scale for Adolescents and Adults (BESAA; [48] for use in Colombian and Nicaraguan participants. The BESAA measures body satisfaction/dissatisfaction and contains 23 items. It was originally developed and validated in adolescent and adult samples in Canada and has three subscales [48].

The subscale ‘*appearance’* (e.g., “I like what I look like in pictures”) takes a holistic view of body image by including multiple aspects related to appearance, without specifying a particular appearance ideal. Therefore, this scale is well suited to use in different cultural contexts where focus lies more on body shape than body size (e.g., [1, 71]), or where other aspects of appearance (e.g., skin colour or hair texture, [21, 41]) may be salient aspects influencing body satisfaction.

The second subscale focuses on satisfaction with ‘*weight’,* with items such as “I am satisfied with my weight”. Higher BMI is a risk factor for body dissatisfaction, which has been confirmed in adults and adolescents around the world [78], including Colombian adults [25]. This subscale therefore assesses a relevant aspect influencing body satisfaction/dissatisfaction.

Items in the '*attribution* subscale' (e.g., “My looks help me to get dates”) assess perceived judgments of others about appearance and weight. However, some scholars chose to exclude this subscale (e.g., [24, 45]), since evaluation from others is not a core aspect of body image and the subscale often showed lower reliability compared to the other two subscales of the BESAA (e.g., [17, 32, 48]).

The BESAA has been translated and validated in Icelandic [32], Italian [17], French [61, 74], Turkish [2], and Portuguese [65]. The English version has also been adapted to different cultural contexts in urban India [24] and Singapore [13]. The BESAA has been translated into Argentinian Spanish by Forbes et al. [22], but its psychometric validity was only assessed ten years later in adolescents in Spain [4]. None of the validation studies described were able to reproduce the original factor structure including all 23 items. Several studies reproduced the original three-factor structures, but excluded many items due to low loadings or high cross-loadings (Turkey: [2], Spain: [4],Italy: [17], Iceland: [32]. In India, France and Brazil, new structures emerged including two-, three-, and four-factor solutions (see [24, 61, 65, 74]).

Across these studies, researchers found good internal reliability for the whole scale and (varying) subscales, temporal stability, and validity (see [4] for a summary of validation studies). However, attempts to force an underlying factor structure have resulted in a varying number of items being retained across samples, which suggests that the BESAA factor structure might be unstable across samples and cultures.

### Study aim

Given the points above, we evaluated a culturally adapted, Latin-American Spanish version of the BESAA (BESAA-LA) retaining as many items as possible, and focused on the assessment of the scale’s reliability, validity, and stability in two different samples within Latin America; university students from urban Colombia and adults from rural Nicaragua.

In Study 1, we present a full validation of the BESAA-LA following the guidelines for translation and validation of body image measures [69, 70]. Our psychometric evaluation of the scale included exploratory factor analysis (EFA), confirmatory factor analysis (CFA) and exploratory structural equation model (ESEM) to assess model fit and factor structure in Colombian participants. We also assessed reliability, validity, temporal stability and gender invariance in this sample.

In Study 2, we tested the generalisability of the scale by assessing internal reliability, validity and model fit of the BESAA-LA in a sample of adults from the Caribbean coast of Nicaragua. Additionally, we assessed measurement invariance across our Colombian and Nicaraguan samples.

## Study 1—validation in Colombia

### Methods

#### Participants

A total of 525 participants completed the questionnaire (*M*_age_ = 24.62, *SD* = 9.95), exceeding the recommended minimum 1:20 item participant ratio (see [29, 69]). Participants were asked about their gender identity, answer options included ‘female’, ‘male’, ‘other’ (with the option to specify) and ‘prefer not to say’. 65% of participants identified as female, 34% as male and 0.6% as other/preferred not to say).

### Procedure

This project complied with ethical guidelines for research with human participants and received ethical approval from Durham University (PSYCH-2020–08-20T11:47:35-dfls13) and Universidad del Norte (Nº 210). All data were collected using an online Questionnaire on Qualtrics™.

Undergraduate psychology students from Universidad del Norte and Corporación Universitaria Reformada in Barranquilla were invited to fill out the questionnaire. Additionally, they received a maximum of 0.5 percentage-point on their final exam when they recruited five people to participate. Out of 697 people who opened the questionnaire, 525 participants (64%) answered all questions. Two weeks later, all participants who completed the questionnaire received an email inviting them to complete the questionnaire again. Due to low participation rates for retest at the two-week interval, participants received another invitation four weeks later. Only 16% of participants (*N* = 84) filled out the questionnaire a second time.

#### Measures

##### Body Esteem Scale for Adolescents and Adults

The Body Esteem Scale for Adolescents and Adults [48] assesses body satisfaction/dissatisfaction and includes 23 items. It is answered on a Likert-scale from 1 = never to 5 = always, with higher average scores indicating higher body satisfaction.

Professor Beverley Mendelson granted permission to translate the Body Esteem Scale for Adolescents and Adults [48] in January 2022. The translation and cross-cultural adaptation followed the guidelines by Swami and Barron [69] and Kling et al. [35]. First, items were translated by two bilingual speakers into Spanish (FA, TT). The scale was then back translated into English by a Colombian bilingual speaker (AC). Any disagreements between translations were resolved through discussion.

In the next step, a committee consisting of two authors (JT, LF) and five students in Colombia confirmed that all wording was culturally appropriate (as suggested by [7]. Feedback on this final translation was then sought from a small group of Colombian students (*N* = 24). All items were judged to be comprehensible, and no further changes were made.

#### Body appreciation

The Latin Spanish version of the Body Appreciation Scale-2 (BAS-2; [26, 72]) was used to measure body appreciation. It includes 10 items on a Likert scale from 1 = never to 5 = always (e.g., “I respect my body”). Higher average scores indicate higher body appreciation. The scale showed good reliability in our sample (Cronbach’s α = 0.94). This scale has been used to test the nomological validity of the BESAA in other validation studies (see, e.g., [4, 28]) and we expected positive correlations between BAS-2 and BESAA-LA scores.

#### Media internalization and sociocultural pressures

The Spanish version of the Sociocultural Attitudes Towards Appearance Scale-4 (SATAQ-4; [40, 64]) was used to measure internalization and sociocultural pressures. The scale consists of five subscales, measuring internalization of the thin ideal, athletic ideal, and sociocultural pressures from family, friends, and the media. Participants respond on a Likert scale, where 1 = strongly disagree and 5 = strongly agree, to statements such as “It is important for me to look athletic”. Higher average scores indicate higher internalization and sociocultural pressures. Cronbach’s alpha confirmed good reliability in our sample with 0.92 (Cronbach’s α). The SATAQ has been used in another validation study to test discriminant validity [4]. We expected negative correlations between the SATAQ-4 subscales and the BESAA-LA.

#### Eating restraint

The restrained eating subscale of the Spanish version of the Eating Disorder Examination Questionnaire (EDE-Q; [42, 54]), was used to assess eating disorder symptoms. This subscale contains 5 items and measures the frequency of eating restraint over the last four weeks on a response scale from 0 = no days to 6 = every day. Higher average scores indicate higher eating disorder symptomatology. Good reliability was confirmed in our sample (Cronbach’s α = 0.86). The EDE-Q has been used in prior studies to test discriminant validity (e.g., [4, 24]). We expected a negative correlation between eating restraint and BESAA-LA.

#### Statistical analysis

Analyses were conducted in RStudio 4.0.3 (RStudio Team 2020). Packages Lavaan [60], and SEM [23] were used for confirmatory factor analysis (CFA) and Exploratory Factor Analysis (EFA). Exploratory structural Equation model (ESEM) was conducted in Mplus version 8.10 [52]. Analytic code and redacted-anonymized data are available on OFS (https://osf.io/q9mj2/).

The fit of previously validated factor structures (i.e., original, Spanish short, Brazilian, and French male and female versions) was first assessed using confirmatory factor analysis (CFA). As these factor structures did not satisfactorily fit our data, exploratory factor analysis (EFA) with a random half of the data was conducted. Parallel analysis [30], the Guttman-Kaiser criterion [27, 33], and the scree plot were considered to decide the number of factors to retain during EFA. Factors needed to fulfil the following criteria (as suggested by [69]): (a) each factor needed to contain at least 3 items, (b) the factor loading for each item needed to be at least 0.35, (c) each factor needed to explain at least 5% of the total variance, (d) items with cross-loadings of > 0.33 on more than one factor would be excluded. The newly found structure was confirmed using CFA on the second half of the data. We performed oblique rotation and used robust weighted least squares estimator (WLSMV), as recommended for categorical variables [51, 56]. Additionally, exploratory structural equation models (ESEM) were used to test model fit. Due to the presence of cross-loadings, ESEM has advantages over CFA since it does not force factor loadings on secondary factors to 0 [56]. A target rotation was specified based on the factor structure derived from EFA. This means we specified on which factor we expected items to load, while not restricting cross-loadings to 0. We followed the code provided by Prokofieva et al. [56].

The following fit indices were used to test adequate model fit, as recommended by Hu and Bentler [31]: Root Mean Square Error of Approximation (RMSEA, values less than 0.06 are considered good, and values between 0.07 and 0.10 are acceptable); Standardized Root Mean Square Residual (SRMR; values below 0.08 are good, values between 0.09 and 0.10 are acceptable); Tucker-Lewis Index and Comparative Fit Index (TLI and CFI; values higher than 0.95 suggest close fit for both, values between 0.90 and 0.94 are acceptable).

Internal consistency was assessed using Cronbach’s alpha coefficient (α; [20]) and McDonald’s Omega (ω; [44]). Alpha and omega values above 0.8 indicate good reliability (see [38]). Convergent and discriminant validity were evaluated using Pearson correlations. Test–retest reliability was assessed using the intraclass correlation coefficient (ICC; [3]), for which values above 0.75 are considered acceptable [55].

A series of multi-group CFA models was performed to assess configural, metric and scalar invariance across men and women—this is to say, whether the general factor structure is the same, whether the specific loadings are equal, and whether they have equal intercepts, respectively. This process involves sequentially creating three models, with every model adding more constraints compared to the previous one. The fit of the models is compared and if fit does not deteriorate in the more constrained model, invariance at the given level can be assumed. To confirm invariance, the chi-square difference test should be nonsignificant and differences in CFI, RMSEA and SRMR between the two models (configural vs. metric; metric vs. scalar) should remain small (ΔCFI < 0.01 and ΔRMSEA < 0.015 or ΔSRMR < 0.030 are considered sufficient, [14]. However, the chi-square difference test is sensitive to sample size (see [69]) and as long as the differences in CFI, RMSEA and SRMR remain small, invariance can still be assumed.

### Results

#### Confirmatory factor analysis with previously validated factor structures

The data did not show multivariate normality (skewness *p* < 0.001, kurtosis *p* = 0.39), as assessed by the Mardia test [43]. A table with descriptive statistics is provided in Additional file 1: S1. None of the previously validated factor structures (original, French female and male versions, Brazilian version, and Spanish short version) showed adequate fit for our data, with the Spanish short version showing closest fit compared to the other factor structures (see Table 1).

**Table 1:** Goodness of fit indices from confirmatory factor analysis using factor structures of other validation studies

	Items	χ2	df	RMSEA	CFI	TLI	SRMR
Original structure	23	123.87	227	0.0984	0.790	0.766	0.079
French female	22	1334.55	206	0.093	0.761	0.732	0.077
French male	21	886.32	183	0.086	0.807	0.778	0.067
Brazilian version	18	618.35	132	0.084	0.832	0.806	0.069
Spanish short	14	327.42.98	74	0.081	0.877	0.849	0.060

CFA with factor structures found in other validation studies (*N* = 525); χ2 = chi-square; df = degrees of freedom; RMSEA = Root Mean Square Error of Approximation; CFI = Comparative Fit Index; TLI = Tucker-Lewis Index; SRMR = Standardized Root Mean Residual

#### Exploratory factor analysis

Exploratory factor analysis was performed with a random half of the sample (*N* = 275). Both parallel analysis and Kaiser-Guttmann criterion (3 eigenvalues were greater than 1; 9.46, 2.26, 1.42; fourth eigenvalue = 0.70) suggested a three-factor structure. In addition, the fit of the unidimensional model was assessed (see Table 3). The three-factor solution showed best fit for the data, according to relative and absolute fit indices. Item 5 (“I think my appearance would help me get a job”) showed poor factor loading and was subsequently removed*.* Four items (Item 4 “I am preoccupied with trying to change my body weight”; item 13 “My looks upset me”; item 17 “I feel ashamed of how I look.”; item 18 “Weighing myself depresses me”) showed loadings above 0.37 on more than one factor and were therefore excluded. The subscales of the 18-item version were named ‘*appearance-positive’*, ‘*appearance-negative’* and ‘*weight’*. All factor loadings derived from EFA are shown in Table 2. CFA with the second random half of the sample showed acceptable fit of the 3-factor version with 18 items (see Table 3 for all results).

**Table 2:** Factor loadings of the 18-item version of the BESAA-LA derived from EFA, with item labels in Spanish and English

Nb	Item Spanish	Item English	Weight	Appearance-negative	Appearance-positive
8	Estoy satisfecho/a con mi peso	*I am satisfied with my weight*	**0.845**	0.137	0.004
10	Estoy a gusto con mi peso	*I really like what I weigh*	**0.836**	0.045	0.082
16	Siento que mi peso es adecuado para mi estatura	*I feel I weigh the right amount for my height*	**0.722**	-0.072	0.127
7	Hay muchas cosas que cambiaría de mi apariencia física si pudiera	*There are lots of things I’d change about my looks if I could*	-0.030	**0.608**	0.223
9	Desearía tener un mejor físico	*I wish I looked better*	0.018	**0.480**	0.162
11	Me gustaría verme como otra persona	*I wish I looked like someone else*	-0.183	**0.602**	0.309
19	Mi peso me hace infeliz	*My weight makes me unhappy*	0.160	**0.396**	0.057
21	Me preocupa mi apariencia física	*I worry about the way I look*	-0.024	**0.571**	-0.014
1	Me gusta cómo me veo en fotos	*I like what I look like in pictures*	-0.066	0.040	**0.580**
2	Otras personas me consideran atractivo/a	*Other people consider me good looking*	-0102	-0.287	**0.805**
3	Estoy orgulloso/a de mi cuerpo	*I am proud of my body*	0.034	0.166	**0.716**
6	Me gusta lo que veo cuando me miro en el espejo	*I like what I see when I look in the mirror*	0.70	0.104	**0.706**
12	A la gente de mi edad le gusta mi apariencia	*People my own age like my looks*	-0.016	-0.160	**0.826**
14	Soy tan atractivo/a como la mayoría de la gente	*I’m as nice looking as most people*	-0.195	0.005	**0.895**
15	Estoy bastante feliz con cómo me veo	*I’m pretty happy about the way I look*	0.082	0.269	**0.659**
20	Mi apariencia me ayuda a conseguir citas	*My looks help me to get dates*	0.013	-0.292	**0.695**
22	Pienso que tengo un buen cuerpo	*I think I have a good body*	0.228	-0.005	**0.681**
23	Me veo tan atractivo/a como me gustaría	*I’m looking as nice as I’d like to*	0.018	0.112	**0.793**

Standardized loadings of exploratory factor analysis, random half of Colombian sample (*N* = 275), highest loadings are shown in bold

**Table 3:** Fit indices from EFA, CFA and ESEM for the 23- and 18-item versions of the BESAA-LA

	Items	χ2	df	RMSEA	CFI	TLI	SRMR
EFA 1-factor	23	725.91	230	0.089	0.706	0.677	0.101
EFA 3-factor	23	278.45	187	0.042	0.946	0.927	0.034
EFA 1-factor	18	522.13	135	0.103	0.715	0.677	0.096
EFA 3-factor	18	155.67	102	0.044	0.960	0.941	0.031
CFA 3-factor	18	326.15	132	0.077	0.893	0.876	0.63
ESEM 3 factor	18	470.70	102	0.083	0.981	0.972	0.027

EFA with random half of the sample (*N* = 275), CFA with the other random half of the sample (*N* = 251), ESEM analysis conducted in Mplus with whole sample (*N* = 525); χ2 = chi-square; df = degrees of freedom; RMSEA = Root Mean Square Error of Approximation; CFI = Comparative Fit Index; TLI = Tucker-Lewis Index; SRMR = Standardized Root Mean Residual

#### ESEM

To confirm factor structure, ESEM analysis was conducted using target rotation (based on EFA structure) in Mplus. Fit indices improved compared to CFA analysis and indicated good fit of the factor structure with the three subscales *‘appearance-positive’,* ‘*appearance-negative’ and ‘weight’*. See Table 3 for fit indices and Additional file 2: S2 for all factor loadings derived from ESEM.

#### Reliability and validity

The 18-item BESAA-LA and all subscales showed good internal reliability (total: α = 0.92, ω = 0.94; ‘*appearance-positive*’: α = 0.92, ω = 0.94; ‘*appearance-negative’*: α = 0.74, ω = 0.78 and ‘*weight*’: α = 0.89, ω = 0.90).

Convergent and discriminant validity were assessed using zero-order correlations. BAS, EDE, SATAQ thin-ideal internalization, athletic internalization and pressures all showed significant correlations with BESAA-LA total scale and subscales in the predicted directions. The BAS showed a strong significant positive correlation with BESAA-LA and all its subscales, confirming convergent validity. Strong significant negative correlations of the BESAA-LA with the thin internalization and pressure subscales of SATAQ, as well as small significant negative correlations with the subscale athletic internalization (except for the positive and weight subscales) confirmed discriminant validity. Eating restraint showed a moderate significant negative relationship with BESAA-LA. See Table 4 for all results.

**Table 4:** Zero-order correlations coefficients (Pearson’s *r*) between BESAA-LA, BAS, SATAQ and EDE

	BESAA-LA-total	BESAA-LA-positive	BESAA-LA-negative	BESAA-LA-weight
BAS	0.79**	0.77**	0.62**	0.50**
SATAQ	− 0.52**	− 0.38**	− 0.57**	− 0.40**
Thin internalization	− 0.46**	− 0.32**	− 0.52**	− 0.37**
Athletic internalization	− 0.12**	− 0.06	− 0.22**	− 0.07
Pressure	− 0.50**	− 0.39**	− 0.50**	− 0.39**
EDE	− 0.42**	− 0.29**	− 0.44**	− 0.41**

**Indicates *p* < 0.01. BESAA-LA = Latin-American Spanish Body Esteem Scale for Adolescents and Adults, subscales appearance-positive, appearance-negative and weight, BAS = Body Appreciation Scale, SATAQ = Sociocultural Attitudes Towards Appearance Questionnaire, subscales thin ideal internalization, athletic internalization and pressures from family, friends and media, EDE = Eating Disorder Examination, subscale eating restraint

#### Temporal stability

Although only 84 participants (16% of our original Colombian sample) responded to the questionnaire a second time, intra-class correlation analysis suggested good test–retest reliability ICC = 0.754, *p* < 0.001, 95%CI 0.645 to 0.833, providing some support for the temporal stability of the BESAA-LA. ANOVAs indicated that those participants did not significantly differ from non-completers in terms of age (*F*(1, 523) = 2.908, *p* = 0.089), gender (*F*(1, 523) = 0.75, *p* = 0.387), or baseline BESAA-LA scores (*F*(1, 523) = 0.177, *p* = 0.674).

#### Measurement invariance across gender

A series of multi-group CFAs were conducted to check for invariance across gender. Table 5 shows the fit of the model for men and women followed by tests of configural, metric and scalar invariance. The configural invariance test confirmed that the factor structure does not differ between men and women. Metric invariance uses a more restricted model with equal factor loadings between groups. The chi-square difference test between the configural and metric model was nonsignificant (*p* = 0.195) and fit indices showed minimal changes, indicating metric invariance. To test scalar invariance, a model with equal intercepts imposed for men and women was created. Compared to the metric model, chi-square difference test was significant (*p* < 0.001), but changes in CFI, RMSEA and SRMR remained small, confirming scalar invariance of the BESAA-LA in men and women in Colombia (see Table 5).

**Table 5:** Configural, metric and scalar measurement invariance of the BESAA-LA across men and women in Colombian sample

	χ2	df	CFI	RMSEA	SRMR	ΔCFI	ΔRMSEA	ΔSRMR
Women	386.48	132	0.885	0.075	0.061			
Men	216.89	132	0.903	0.060	0.065			
Configural	585.25	264	0.999	0.053	0.059			
Metric	472.57	279	0.995	0.054	0.065	− 0.003	0.001	0.005
Scalar	521.97	294	0.993	0.057	0.068	− 0.003	0.003	0.003

Multi-group CFA across women (*N* = 341) and men (*N* = 178); χ2 = chi-square; df = degrees of freedom; RMSEA = Root Mean Square Error of Approximation; CFI = Comparative Fit Index; SRMR = Standardized Root Mean Residual

### Interim discussion

The aim of Study 1 was to translate and culturally adapt the Body Esteem Scale for Adolescents and Adults in a Colombian adult sample. The 18-item BESAA-LA showed good internal reliability, temporal stability and validity. As expected, the BESAA-LA showed a positive correlation with body appreciation, a construct that is related to body satisfaction, but that incorporates a broader range of factors, such as body functionality and self-love [73]. Significant moderate negative correlations were shown with the SATAQ subscales thin ideal internalization and sociocultural pressures, whereas the athletic internalization subscale showed significant, but small negative correlations with the BESAA-LA. This is in line with other studies that found lower correlations between the BESAA and athletic subscale, compared to other SATAQ subscales [4].

When assessing factorial validity, the original three-factor structure could not be reproduced. However, our three factor solution with subscales ‘*appearance-positive’, ‘appearance-negative’* and ‘*weight’* resembles the Brazilian structure [65]. This resemblance is not surprising, as these Latin American countries share geographical proximity, as well as a focus on appearance and beauty, which is reflected in high incidence of plastic surgery per capita in both Colombia and Brazil (International Association for Aesthetic/Cosmetic surgery survey 2020). Conceptually, the two subscales ‘*appearance-positive’ and ‘appearance-negative’* support the proposition that positive and negative body image might be two separate constructs, rather than the ends of a continuum [65, 77]. The third subscale ‘*weight’* has been found consistently across all validation studies conducted so far (Original validation [48], Iceland [32], Italy [17], Turkey [2], India [24], Spain [4] and Brazil [65]).

One item (item 5; “I think my appearance would help me get a job”) showed poor factor loading and was therefore excluded. This is in line with the French versions for men and women, where this item was excluded due to poor factor loading [61, 74]. Four items were excluded due to high cross-loadings. Items 4 and 18 ("I am preoccupied with trying to change my body weight” and “Weighing myself depresses me”) loaded on the ‘*weight’* and ‘*appearance-negative’* subscales, which makes conceptual sense, as both items are negative statements referring to weight. On the other hand, items 13 and 17 (“My looks upset me” and “I feel ashamed of how I look”) loaded both on ‘*appearance-positive’* and ‘*appearance-negative’* subscales. This could be due to the nature of these two items; whereas most items in the ‘*appearance-negative*’ subscale focus on wanting to look better or like someone else, these two items assess specific negative emotional reactions to one’s own physical appearance. During data collection, several participants commented that these questions had felt unusually emotionally intrusive compared to the rest of the questionnaire, which may explain the unusual loading pattern.

## Study 2—evaluation in Nicaragua

In order to check the reliability, validity and factor structure of the BESAA-LA, we administered it in a second Spanish speaking adult population, to adults in rural Nicaragua.

### Methods

#### Participants

102 participants (*M*_age_ = 22.16, *SD* = 4.72; 73% women, 27% men) completed the questionnaire. Among the participants, 49% identified as Mestizo, 38% as belonging to an afro-descended ethnic group (Creole or Garifuna), 8% to an indigenous ethnic group (Miskitu or Ulwa), and 5% identified as ‘other’ (but did not specify their ethnic identity). For a detailed description of ethnic groups in this region, see Boothroyd et al. [5] and Thornborrow et al. [71].

#### Procedure

Ethical approval for data collection was given by Durham university (PSYCH-2020–08-20T11:47:35-dfls13). There is no formal ethics committee at URACCAN (Universidad de las regiones autónomas de la costa caribe Nicaragüense) where the data were gathered, however we received approval from the university’s vice rector to collect data. We sought feedback on the questionnaire items from WB and two local students, who confirmed congruency with local (Nicaraguan) terminology. The anonymous online questionnaire was distributed via Qualtrics™ to undergraduate psychology students and adults through personal contacts in the Pearl Lagoon region on the Caribbean Coast of Nicaragua.

#### Measures

As in Study 1, we used our translated version of the Body Esteem Scale for Adolescents and Adults (BESAA-LA) and the Latin Spanish version of the Body Appreciation Scale-2 (BAS-2; [26, 72]). The BAS-2 showed good reliability in our Nicaraguan sample (Cronbach’s α = 0.95).

#### Media internalization

To measure media internalization, we used the Spanish version of the Sociocultural Attitudes Towards Appearance Questionnaire-3 (SATAQ-3; [8, 62]). Participants respond to such items as “I would like my body to look like the people who are in movies”) on a 5-point Likert scale ranging from “strongly disagree” to “strongly agree”. Higher average scores indicate greater media internalization. Based on earlier work in this part of Nicaragua (see [71]), negatively worded items were removed. Items about magazines (which are not locally available) were adapted to refer to social media (e.g., “social media is an important source of information about fashion and ‘being attractive’”). The adapted scale showed good reliability in our sample (Cronbach’s α = 0.94).

#### Statistical analysis

As in Study 1, we used Cronbach’s alpha (α; [20]) and McDonald’s Omega (ω; [44]) to assess internal consistency, and Pearson correlations to assess validity. As described in Study 1, we performed confirmatory factor analysis to assess factor structure and multi-group CFA models to assess measurement invariance across Colombian and Nicaraguan participants.

### Results

#### Psychometric analysis using CFA

Goodness of fit indices from CFAs using the Latin-American Spanish version, the original factor structure, French male and female, Brazilian, and Spanish short versions revealed that the Latin-American Spanish version found in Colombia showed the best fit. See Table 6 for all fit indices.

**Table 6:** Goodness of fit indices from confirmatory factor analysis using factor structures of validation studies

	Items	χ2	df	RMSEA	CFI	TLI	SRMR
Latin American Spanish	18	185.03	132	0.063	0.910	0.896	0.070
Original structure	23	394.64	227	0.086	0.871	0.856	0.086
French female	22	356.46	206	0.093	0.883	0.869	0.076
French male	21	302.31	183	0.080	0.901	0.886	0.076
Brazilian version	18	214.31	132	0.079	0.913	0.899	0.080
Spanish short	14	123.10	74	0.081	0.928	0.912	0.089

CFA with previously validated factor structures in Nicaraguan participants (*N* = 102); Χ^2^ = chi-square; df = degrees of freedom; RMSEA = Root Mean Square Error of Approximation; CFI = Comparative Fit Index; TLI = Tucker-Lewis Index; SRMR = Standardized Root Mean Residual

#### Reliability and validity

Internal consistency was good for the BESAA-LA total scale (α = 0.93, ω = 0.95) and the subscales ‘*appearance-positive’* (α = 0.88, ω = 0.93), ‘*appearance-negative’* (α = 0.80, ω = 0.84) and ‘*weight’* (α = 0.91, ω = 0.91). Validity of the BESAA-LA was confirmed through positive correlations of the total BESAA-LA and subscales with BAS, as well as negative correlations with SATAQ subscales. See Table 7 for all results.

**Table 7:** Zero-order correlations coefficients (Pearson’s *r*) between BESAA-LA, BAS and SATAQ

	BESAA-LA-total	BESAA-LA-positive	BESAA-LA-negative	BESAA-LA-weight
BAS	0.87**	0.85**	75**	0.69**
SATAQ total	− 0.60**	− 0.52**	− 0.62**	− 0.49**
General internalization	− 0.63**	− 0.51**	− 0.70**	− 0.48**
Athletic internalization	− 0.5**	− 0.42**	− 0.55**	− 0.44**
Pressure	− 0.55**	− 0.48**	− 0.56**	− 0.44**

**Indicates *p* < 0.01. BESAA-LA = Latin-American Spanish version of the Body Esteem Scale for Adolescents and Adults, subscales appearance-positive, appearance-negative and weight, BAS = Body Appreciation Scale, SATAQ = Sociocultural Attitudes Towards Appearance Questionnaire, subscales general internalization, athletic internalization and pressure

#### Measurement invariance across countries

Multi-group CFAs confirmed scalar invariance across Colombia and Nicaragua. Even though the chi-square difference test was significant when comparing the metric and scalar models ( χ2 *p* < 0.001), differences in CFI, RMSEA and SRMR remained small after the fixation of loadings and intercepts and scalar invariance can therefore be assumed (see Table 8).

**Table 8:** Configural, metric and scalar measurement invariance of the BESAA-LA across country

	χ2	df	CFI	RMSEA	SRMR	ΔCFI	ΔRMSEA	ΔSRMR
Colombia	528.20	153	0.874	0.0761	0.058			
Nicaragua	185.03	132	0.910	0.063	0.070			
Configural	682.72	264	0.997	0.054	0.057			
Metric	582.03	279	0.984	0.068	0.070	− 0.0113	0.0015	0.013
Scalar	620.88	294	0.982	0.069	0.072	− 0.001	0.001	0.002

Multi-group CFA across Colombia (*N* = 526) and Nicaragua (*N* = 102); χ2 = chi-square; RMSEA = Root Mean Square Error of Approximation; CFI = Comparative Fit Index; SRMR = Standardized Root Mean Residual

## Discussion

The present study aimed to assess the reliability, validity, stability, measurement invariance, and factor structure of a culturally and linguistically adapted version of the Body Esteem Scale for Adolescents and Adults in Latin America. The 18-item Latin-American Spanish version of the BESAA (BESAA-LA) showed good reliability (measured by Cronbach’s alpha and omega) and validity (using BAS and SATAQ) across two different Spanish speaking adult samples from urban Colombia and rural Nicaragua.

For both samples, none of the existing factor structures found in other validation studies (original structure, French male and female versions, Brazilian, and Spanish short versions) showed adequate fit. A three-factor structure established through EFA consisted of the three subscales ‘*appearance-positive’*, ‘*appearance-negative’* and ‘*weight’,* and was confirmed through CFA in both Colombian and Nicaraguan samples. Importantly, the factor structure resembles the structure found with adolescents in Brazil and showed invariance across Colombia and Nicaragua, which suggests it may be suitable across Latin American populations.

However, we also note that there is a general difficulty in reproducing factor structures of the BESAA consistently in the broader literature, and that lower internal reliability was found for subscales than the full scale for our own samples and in other studies [2, 17, 48]. These points suggest that it might be better to use the BESAA as a holistic measure of body image, and that researchers should generally use total scale scores in their studies, rather than the individual subscales, unless there is a strong reason to isolate one aspect.

### Strengths and limitations

This study benefits from a rigorous approach to scale adaptation, following the translation and cultural adaptation processes proposed by Swami and Barron, [69] and Kling et al. [35]. Our dataset in Colombia was large, and locally validated measures (SATAQ-4 and BAS) were used to confirm validity. Furthermore, this is the first formal validation study of a body image measure in a Nicaraguan population. Evidence of increasing body image risks in that country point to the potential utility of such a scale [71].

Additionally, multi-group CFA confirmed structural, metric, and scalar invariance of the scale across men and women (in Colombia) and across Colombian and Nicaraguan participants. This is the first study to assess measurement invariance of the BESAA-LA across different countries. Partial invariance across gender was established in Brazilian adolescents [65], whereas other studies did not assess measurement invariance of the scale at all. Evidence of measurement invariance is an important condition to conduct comparisons of scores between different groups and confirms that differences in scores are results of attitudinal, not psychometric differences of study populations [14, 15].

Data in Colombia were collected in two different universities (one private, one public) in Barranquilla. This allowed us to have a socioeconomically diverse sample of urban participants. Additionally, we tested the BESAA-LA in a sample from rural Nicaragua. Body dissatisfaction and body ideals often differ between urban and rural regions [6, 70], as well as higher and lower SES groups [25]. The BESAA-LA appears to be psychometrically sound in samples from both urban and rural contexts. This suggests that the scale may be suited to a variety of contexts.

Although a very small number of respondents limits the reliability of our estimates [55], the scale appears to have shown good temporal stability (as measured by ICC) in a subsample of our Colombian participants. Further supporting this point, at the initial assessment, test–retest ‘responders’ did not differ statistically from ‘non-responders’ in terms of gender, age or initial BESAA-LA scores. However, we aimed for an interval of two weeks between test and retest (as suggested by [55]), but were only able to reach a sufficient retest sample size within six weeks after initial assessment. Although this is not ideal, we deemed it better to have a retest interval of two to six weeks than not doing any retest analysis at all. Also, the considerably smaller size of the Nicaraguan sample, although sufficient to assess reliability, validity and conduct CFA (according to [34]), did not meet the ideal participant-to-item ratio of 20:1 (see [69]) and the CFA in the Nicaraguan sample should therefore be interpreted with caution.

Finally, the BESAA-LA proved to be a good tool for research with adults in these two regions, however future studies are needed to also assess the psychometric properties of the BESAA-LA in other age groups, for example adolescent populations, and in other Latin American samples.

## Conclusion

The Latin-American Spanish Version of the Body Esteem Scale for Adolescents and Adults (BESAA-LA) showed good psychometric properties and appeared to be a valid tool to assess body satisfaction/dissatisfaction in two distinct adult populations in Spanish speaking Latin American countries.

### Supplementary Information

**Additional file 1:** Descriptive statistics.

**Additional file 2:** ESEM results.

## Data Availability

Anonymised data and R code are available at: https://osf.io/q9mj2/.
