# Supplementary material for: Validation of a Latin-American Spanish version of the Body Esteem Scale for Adolescents and Adults (BESAA-LA) in Colombian and Nicaraguan adults
Source: J Eat Disord. 2023 Dec 8;11:219. doi: 10.1186/s40337-023-00942-5 (PMC10709846; doi:10.1186/s40337-023-00942-5)
Supplement: Supplementary file 1 — Additional file 1: Descriptive statistics. [file 40337_2023_942_MOESM1_ESM.docx]

|  | **N** | **Mean** | **SD** | **Median** | **Trimmed** | **Mad** | **Min** | **Max** | **range** | **skew** | **kurtosis** | **se** |
| --- | --- | --- | --- | --- | --- | --- | --- | --- | --- | --- | --- | --- |
| BESAA1 | 526 | 2.40 | 0.97 | 2 | 2.41 | 1.48 | 0 | 4 | 4 | -0.19 | -0.25 | 0.04 |
| BESAA2 | 525 | 2.64 | 0.84 | 3 | 2.66 | 1.48 | 0 | 4 | 4 | -0.32 | 0.03 | 0.04 |
| BESAA3 | 526 | 2.68 | 1.01 | 3 | 2.74 | 1.48 | 0 | 4 | 4 | -0.33 | -0.51 | 0.04 |
| BESAA4_recoded | 526 | 2.13 | 1.25 | 2 | 2.16 | 1.48 | 0 | 4 | 4 | -0.21 | -0.96 | 0.05 |
| BESAA5 | 525 | 1.99 | 1.28 | 2 | 1.99 | 1.48 | 0 | 4 | 4 | -0.04 | -1 | 0.06 |
| BESAA6 | 526 | 2.65 | 0.92 | 3 | 2.71 | 1.48 | 0 | 4 | 4 | -0.36 | -0.18 | 0.04 |
| BESAA7_recoded | 526 | 1.80 | 1.27 | 2 | 1.75 | 1.48 | 0 | 4 | 4 | 0.02 | -1.05 | 0.06 |
| BESAA8 | 526 | 2.20 | 1.23 | 2 | 2.25 | 1.48 | 0 | 4 | 4 | -0.18 | -0.93 | 0.05 |
| BESAA9_recoded | 526 | 1.23 | 1.18 | 1 | 1.11 | 1.48 | 0 | 4 | 4 | 0.58 | -0.60 | 0.05 |
| BESAA10 | 526 | 2.23 | 1.22 | 2 | 2.28 | 1.48 | 0 | 4 | 4 | -0.27 | -0.80 | 0.05 |
| BESAA11_recoded | 526 | 2.73 | 1.21 | 3 | 2.86 | 1.48 | 0 | 4 | 4 | -0.63 | -0.56 | 0.05 |
| BESAA12 | 526 | 2.58 | 0.91 | 3 | 2.63 | 1.48 | 0 | 4 | 4 | -0.33 | -0.11 | 0.04 |
| BESAA13_recoded | 526 | 2.95 | 1.11 | 3 | 3.09 | 1.48 | 0 | 4 | 4 | -0.80 | -0.30 | 0.05 |
| BESAA14 | 525 | 2.58 | 1.02 | 3 | 2.64 | 1.48 | 0 | 4 | 4 | -0.28 | -0.52 | 0.04 |
| BESAA15 | 526 | 2.69 | 1.00 | 3 | 2.76 | 1.48 | 0 | 4 | 4 | -0.39 | -0.53 | 0.04 |
| BESAA16 | 526 | 2.42 | 1.35 | 3 | 2.53 | 1.48 | 0 | 4 | 4 | -0.44 | -0.99 | 0.06 |
| BESAA17_recoded | 526 | 2.96 | 1.06 | 3 | 3.10 | 1.48 | 0 | 4 | 4 | -0.80 | -0.05 | 0.05 |
| BESAA18_recoded | 525 | 2.88 | 1.24 | 3 | 3.06 | 1.48 | 0 | 4 | 4 | -0.90 | -0.25 | 0.05 |
| BESAA19_recoded | 526 | 2.56 | 1.37 | 3 | 2.69 | 1.48 | 0 | 4 | 4 | -0.55 | -0.95 | 0.06 |
| BESAA20 | 525 | 2.10 | 1.24 | 2 | 2.13 | 1.48 | 0 | 4 | 4 | -0.23 | -0.91 | 0.05 |
| BESAA21_recoded | 525 | 1.81 | 1.25 | 2 | 1.77 | 1.48 | 0 | 4 | 4 | 0.27 | -0.85 | 0.05 |
| BESAA22 | 525 | 2.38 | 1.04 | 2 | 2.41 | 1.48 | 0 | 4 | 4 | -0.25 | -0.46 | 0.05 |
| BESAA23 | 525 | 2.16 | 1.13 | 2 | 2.18 | 1.48 | 0 | 4 | 4 | -0.11 | -0.67 | 0.05 |
| BESAA total | 526 | 2.40 | 0.74 | 2.5 | 2.44 | 0.67 | 0.05 | 3.95 | 3.91 | -0.44 | -0.18 | 0.03 |

# Supplementary Material 1

**Table S1.** Descriptive statistics of the Latin-American Spanish Body Esteem Scale for Adolescents and Adults (BESAA-LA)

*Note.* Descriptive statistics in Colombian sample (*N* = 526), using function describe() in R; BESAA = Esteem Scale for Adolescents and Adults, SD = Standard deviation, se = standard error.
