# Supplementary material for: Validation of a Latin-American Spanish version of the Body Esteem Scale for Adolescents and Adults (BESAA-LA) in Colombian and Nicaraguan adults
Source: J Eat Disord. 2023 Dec 8;11:219. doi: 10.1186/s40337-023-00942-5 (PMC10709846; doi:10.1186/s40337-023-00942-5)
Supplement: Supplementary file 2 — Additional file 2: ESEM results. [file 40337_2023_942_MOESM2_ESM.docx]

# Supplementary Material S2 – ESEM results

**Table S2.** Factor loadings of the 18-item version of the BESAA-LA derived from ESEM, with item labels in Spanish and English

| **Nb** | **Item Spanish** | **Item English** | **Weight** | **Appearance-negative** | **Appearance-positive** |
| --- | --- | --- | --- | --- | --- |
| 8 | Estoy satisfecho/a con mi peso. | *I am satisfied with my weight.* | **0.894** | 0.072 | 0.034 |
| 10 | Estoy a gusto con mi peso. | *I really like what I weigh.* | **0.906** | -0.001 | 0.063 |
| 16 | Siento que mi peso es adecuado para mi estatura. | *I feel I weigh the right amount for my height.* | **0.782** | -0.154 | 0.137 |
| 7 | Hay muchas cosas que cambiaría de mi apariencia física si pudiera. | *There are lots of things I’d change about my looks if I could.* | 0.040 | **0.719** | 0.165 |
| 9 | Desearía tener un mejor físico. | *I wish I looked better.* | 0.132 | **0.628** | 0.070 |
| 11 | Me gustaría verme como otra persona. | *I wish I looked like someone else.* | -0.166 | **0.606** | 0.299 |
| 19 | Mi peso me hace infeliz. | *My weight makes me unhappy.* | 0.291 | **0.209** | -0.039 |
| 21 | Me preocupa mi apariencia física. | *I worry about the way I look.* | 0.073 | **0.533** | 0.036 |
| 1 | Me gusta cómo me veo en fotos. | *I like what I look like in pictures.* | -0.069 | 0.050 | **0.656** |
| 2 | Otras personas me consideran atractivo/a. | *Other people consider me good looking.* | -0.113 | -0.398 | **0.925** |
| 3 | Estoy orgulloso/a de mi cuerpo. | *I am proud of my body.* | 0.096 | 0.291 | **0.646** |
| 6 | Me gusta lo que veo cuando me miro en el espejo. | *I like what I see when I look in the mirror.* | 0.078 | 0.255 | **0.646** |
| 12 | A la gente de mi edad le gusta mi apariencia. | *People my own age like my looks.* | -0.032 | -0.239 | **0.884** |
| 14 | Soy tan atractivo/a como la mayoría de la gente. | *I’m as nice looking as most people.* | -0.123 | 0.079 | **0.837** |
| 15 | Estoy bastante feliz con cómo me veo. | *I’m pretty happy about the way I look.* | 0.116 | 0.305 | **0.641** |
| 20 | Mi apariencia me ayuda a conseguir citas. | *My looks help me to get dates.* | -0.005 | -0.276 | **0.763** |
| 22 | Pienso que tengo un buen cuerpo. | *I think I have a good body.* | 0.268 | 0.140 | **0.583** |
| 23 | Me veo tan atractivo/a como me gustaría. | *I’m looking as nice as I’d like to.* | 0.098 | 0.237 | **0.666** |

*Note.* Standardized loadings of Exploratory structural equation model (*N* = 526), run in Mplus; highest loadings are shown in bold.
